# Design of Superhydrophobic Shape Memory Composites with Kirigami Structures and Uniform Wetting Property

**DOI:** 10.3390/polym15183738

**Published:** 2023-09-12

**Authors:** Zhe Zhao, Xinlin Li, Dongsong Wei, Jian Sun, Jinsong Leng

**Affiliations:** 1 Centre for Composite Materials and Structures, Harbin Institute of Technology (HIT), Harbin 150080, China; 21s018054@stu.hit.edu.cn (Z.Z.); lengjs@hit.edu.cn (J.L.); 2 Key Laboratory of Bionic Engineering (Ministry of Education), Jilin University, Changchun 130022, China; weids@jlu.edu.cn

**Keywords:** shape memory composites, kirigami structure, superhydrophobicity, water dynamics

## Abstract

With the continuous increase in human demand to improve aircraft performance, intelligent aircraft technologies have become a popular research field in recent years. Among them, the deformable skin structure has become one of the key technologies to achieve excellent and reliable performance. However, during the service, deformable skin structures may encounter problems such as surface impact and adhesion of droplets in rainy weather or surface icing in low-temperature environments, which can seriously affect the flight safety of the aircraft. One way to overcome these issues is to use superhydrophobic shape memory materials in the structure. In this regard, first, shape memory composites were prepared with shape memory epoxy resin as the matrix and carbon fiber orthogonal woven fabric as the reinforcement material. Superhydrophobic shape memory composites (SSMCs) were then obtained by casting the kirigami composite with superhydrophobic carbon nanotube–polydimethylsiloxane (CNT@PDMS) mixture, and the surface was processed by laser micromachining. Shape memory performance and surface wetting performance were determined by material testing methods. The results showed that the shape memory recovery rate can reach 85.11%, the surface is superhydrophobic, the average water contact angle is 156.9 ± 4.4°, and the average rolling angle is 3 ± 0.5°. The three-point bending test of the specimens with different kirigami cell configurations showed that the shape memory composite based on the rectangular structure has the best deformability with an aspect ratio of 0.4. From the droplet impact test, it was found that the impact speed of water droplets and the curvature of the surface can greatly affect the dynamic performance of water. This work is expected to be of significant research value and importance for developing functional deformable skin materials.

## 1. Introduction

Morphing aircraft [1,2,3] can achieve optimal aerodynamic performance by actively changing their aerodynamic shape according to changes in the flight environment and mission requirements, which is one of the key development areas for future flight equipment. The key structure of aircraft that enables the deformation is the deformable wing skin structure, which can continuously smooth the deformation [4,5,6]. The emergence of smart materials provides an important material foundation for the development of deformable wing skin structures [7,8,9]. Among them, shape memory polymer composites (SMPCs) are noteworthy and are composed of shape memory polymer (SMP) as the matrix and various fibers (such as carbon fiber, glass fiber, and aramid fiber) as the reinforcement [10,11,12]. With its unique variable stiffness properties, SMPC can be used to design and fabricate deformable wing skin structures. Li et al. [13] proposed an integrated deformable/load-bearing skin structure based on the SMPC. They studied the toughness and tear resistance characteristics of SMPC through dynamic mechanical testing, thermodynamic performance testing, and tear resistance testing. It was applied to variable-curvature wings and variable wing tips, confirming the feasibility of using SMPC as a skin. Gong [14] developed an electrically driven SMPC with carbon fiber as the reinforcement material and shape memory epoxy resin as the matrix, obtaining a variable-stiffness corrugated skin. Theoretical, experimental, and finite element studies have shown that the SMPC has superior mechanical properties, shape memory recovery performance, and electrical heating performance. However, in actual working conditions, such as in rainy weather, the performance of the deformable wing skin may be affected by raindrops. When the ambient temperature is below zero, another problem, i.e., the icing of the surface, may occur.

Inspired by the “Lotus effect”, researchers have developed and researched many kinds of superhydrophobic materials for a wide range of applications such as waterproofing, self-cleaning, anti-icing, droplet manipulation, infiltration control, demisting, and drag reduction [15,16,17,18]. When a drop hits a superhydrophobic surface, the drop will produce six different impact phenomena: deposition, prompt splash, crown splash, retraction and fragmentation, partial rebound, and complete rebound [19]. Various phenomena caused by the droplet impact are related to the physical properties of the droplet such as droplet density, droplet viscosity, surface tension, and droplet diameter [20]. In addition, the dynamic performance of impacting droplets is related to surface properties such as surface wettability, surface roughness, and surface temperature [21], and also other impact factors such as the initial impact velocity, and impact angle [22]. Liu et al. [23] showed that surfaces with larger tip angles in microstructures can produce a significant cake-like bouncing, which is characterized by a significantly reduced contact time. Aria et al. [24] developed carbon nanotube superhydrophobic surfaces and conducted droplet impact tests. Their results showed that when the *We* number was low, the droplets rebounded completely. But when the *We* number was at a moderate level, the droplet rebound process produced a Worthington jet and satellite droplets. On the other hand, when the *We* number was high, the droplets broke, and many secondary droplets appeared. In addition, there was no sticking phenomenon during the droplet impact process, which confirmed that the developed superhydrophobic surface had excellent performance. Sahoo et al. [25] studied the effect of liquid droplets on inclined surfaces with experimental methods and found that the maximum spreading coefficient and spreading time decreased with the increase in inclination angle and *We* number. They also investigated the influence of surface tension and liquid viscosity on superhydrophobic surfaces. Guan et al. [26] studied the impact of droplets on an inclined surface using numerical methods, considering various factors such as initial impact velocity, droplet diameter, surface wettability, and inclination angle. They analyzed the effects of these factors on droplet morphology, contact time, spreading time, and maximum dimensionless spreading coefficient after impact. The use of superhydrophobic materials to impart surface anti-wetting performance to shape memory composite materials can effectively reduce the adhesion of surface droplets, reduce the time of droplet impact, or solve the problem of surface icing and improve the reliability of deformable wing skin structures [27].

In this paper, we present a new method to prepare superhydrophobic shape memory composites (SSMCs). Shape memory composite materials were prepared through hot-pressing process, and the shape memory kirigami structures were designed by altering the aspect ratio as the design parameters. Also, the SSMC was finally prepared by compositing with the CNT@PDMS mixture, followed by laser ablation. The results of static and dynamic wettability analysis of the SSMC surface showed that it has uniform superhydrophobic performance and low adhesion. Meanwhile, it exhibited good shape memory and mechanical properties, which allows its potential use as a morphing wing skin. Therefore, this study is expected to provide insight into the design of smart morphing materials with anti-wetting performance.

## 2. Experiment

### 2.1. Materials

Shape memory epoxy resin was chosen as the matrix for preparing shape memory composite materials. In the prepolymer of this epoxy resin, E51 epoxy resin of Phoenix bisphenol A (Nantong Xing Chen Synthetic Material Co., Ltd., Nantong, China) was used. The reinforcing phase of composite materials used in the preparation of shape memory epoxy resin composites in this project was T300-3k carbon fiber orthogonal woven fabric, produced by Dongli Group of Japan. The superhydrophobic modifier was 1H,1H,2H,2H-perfluorooctyl trichlorosilane (PFOTS, Machlin, Shanghai, China) with very low surface energy.

### 2.2. Preparation of Superhydrophobic Shape Memory Composites

#### 2.2.1. Design of Composite Structure

**Figure 1:**
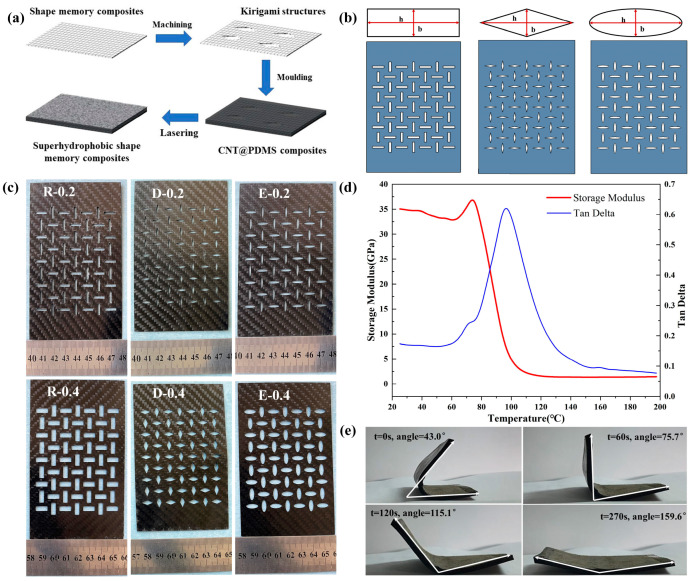
(**a**) Preparation process; (**b**) cell design of shape memory composite with rectangular structure (R), diamond structure (D), elliptical structure (E); (**c**) images of shape memory composites of different kirigami structures with aspect ratio of 0.2 and 0.4; (**d**) shape recovery characteristics; (**e**) dynamic thermomechanical test (DMA) of superhydrophobic shape memory composites.

The whole process of preparing superhydrophobic shape memory composite materials is shown in Figure 1a. E51 epoxy resin and diaminodiphenylmethane (DDM, Machlin, Shanghai, China) curing agent (E51/DDM = 100:15) were added into the beaker and magnetically stirred at a speed of 300 r/min for about 20 min. The precut carbon fiber woven fabric is placed on the molding plate, and the shape memory epoxy resin is evenly coated on the carbon fiber fabric using a pig hair bristle brush to produce epoxy-based composite materials. Adopting the vacuum hot-pressing method, the wetted epoxy-based composites were cured at 80 °C for 2.5 h and then at 150 °C for 2 h. Kirigami structures are usually designed in rectangular, diamond, and elliptical shapes, as shown in Figure 1b. The key parameters of each cell are listed in Table S1. Based on different ratios (b/h), three types of kirigami structures with different aspect ratios were prepared by machining method, as shown in Figure 1c.

#### 2.2.2. Preparation of Superhydrophobic CNT@PDMS Mixture

Multi-walled carbon nanotubes were first modified by 1% wt. of PFOTS in ethanol solution for 6 h. The modified multi-walled carbon nanotubes were then washed with ethanol solution and centrifuged 5 times repeatedly. After that, the obtained multi-walled carbon nanotubes were dried at 80 °C for 3 h. Subsequently, superhydrophobic CNT@PDMS was prepared with a weight ratio of silicone rubber/ethyl acetate/superhydrophobic multi-walled carbon nanotubes/silicone rubber curing agent of 40:18:1:4.

#### 2.2.3. Preparation of Superhydrophobic Shape Memory Composites

The prepared shape memory epoxy-based composites were placed in a polytetrafluoroethylene mold, then the superhydrophobic CNT@PDMS mixture was poured into the mold. Afterward, the mold was transferred to a vacuum oven to discharge the bubbles at room temperature for 30 min. The composites were cured in the mold at 60 °C for 8 h, and the composite plate was obtained after being removed from the mold. Finally, superhydrophobic shape memory composites (SSMCs) were prepared by laser ablation to build the micro textures and expose the superhydrophobic CNTs. The speed of laser processing was 1000 mm/s, the power was 4 W, and the width of the laser was 0.05 mm.

### 2.3. Characterization

#### 2.3.1. Surface Characterization

The surface morphology was investigated with scanning electron microscopy (Thermo QuattroS, Brno, Czech Republic) and 3D optical microscopes (Bruker Contour GT-X, Berlin, Germany). The surface chemicals were evaluated with EDS (EDAX ELECT PIUS, Leicester, UK) and FTIR (Thermo Nicolet iS5, Ashland, VA, USA). Surface wettability was evaluated using a contact angle meter (Shengding-100S, Ningbo, China).

#### 2.3.2. Shape Memory Test

When the sample is heated above the glass transition temperature, it deforms into a temporary U shape under external load. The external force was maintained, the sample was cooled to room temperature, its temporary shape was fixed, and finally the angle of the temporary shape was measured and recorded. Subsequently, the material was placed on a heating table preheated above the glass transition temperature to initiate the shape recovery of the U-shape sample. The entire shape recovery behavior was recorded using a camera, and the shape fixation rate (*R*_f_) and shape recovery rate (*R*_r_) of the material were calculated using Formulas (1) and (2) given below:

(1) $$R_{f}=\frac{180-\theta_{0}}{180}\times 100\%$$

(2) $$R_{r}=\frac{\theta_{t}-\theta_{0}}{180-\theta_{0}}\times 100\%$$

where *θ*_0_ is the bending angle of the temporary shape; *θ*_t_ is the recovery angle when the driving time is t seconds.

#### 2.3.3. Water Dynamics Evaluation

As shown in Figure S3, the droplet impact test system consists of a droplet generation device, a shooting system, a light source, etc. The syringe is fixed on the height-adjustable platform, and, by slowly pressing the syringe, small droplets with a diameter of about 3 mm can be generated. The imaging system includes the Phantom Miro C320 high-speed camera. The detailed parameters of the testing process are listed in Table S2.

## 3. Results and Discussion

### 3.1. Shape Memory Characteristic Testing

Dynamic thermomechanical analysis (DMA, Q800, New Castle, DE, USA) was used to analyze the dynamic thermomechanical properties of shape memory composites. According to the analysis in Figure 1e, the energy storage modulus of the prepared shape memory composite is 35.04 GPa at 25 °C. Also, its loss factor reaches its maximum value at 97 °C and then decreases continuously with increasing temperature. For the superhydrophobic shape memory composite materials, the shape memory recovery test results are shown in Figure 1c. The initial shape of the SSMC was a flat plate. It was heated above the glass transition temperature (*T*_g_ ~ 97 °C) on a 110 °C heating table, and the sample was deformed into a U-shaped sample using an external force. It was then cooled to room temperature to fix its temporary shape, as shown in Figure 1d, with a shape fixation rate of 76.11%. Subsequently, the sample with the temporary shape was placed back on the heating table to undergo shape memory recovery deformation. At 60 s, the shape memory recovery rate was 23.87%, and at 120 s the shape memory recovery rate was 52.63%. Finally, at 270 s, the maximum recovery angle reached 159.6°, at which point the material shape recovery rate reached 85.11%. Therefore, it can be concluded that the SSMC presents good shape memory performance, but its shape memory recovery performance degrades when incorporated with elastic materials. The reason is that the presence of silicone rubber reduces the efficiency of heat transfer and prolongs the recovery time, and, on the other hand, the presence of kirigami structure destroys the continuity of the fiber. In addition, the dislocation of the fiber decreases the shape memory recovery rate of the prepared samples.

### 3.2. Bending Performance of SSMC

**Figure 2:**
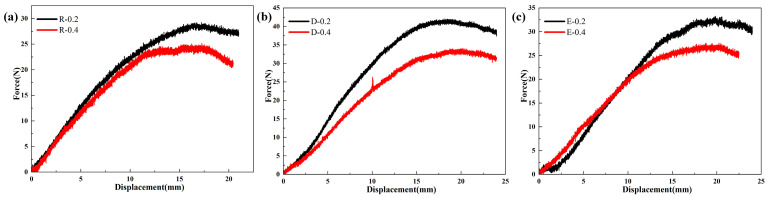
Force–displacement curve of superhydrophobic shape memory composites: (**a**) rectangular structure, (**b**) diamond structure, (**c**) elliptical structure with aspect ratios of 0.2 and 0.4.

In this study, six superhydrophobic shape memory composites with different kirigami structures were considered to investigate the bending properties of these materials. The INSTRON 3382 universal mechanical testing machine (USA) was used to conduct three-point bending tests at room temperature. From the force–displacement curves of SSMC with rectangular kirigami structures of different aspect ratios shown in Figure 2a, it can be seen that the bending loads almost coincide at the beginning and basically maintain a linear increase during the bending deformation process. For sample R-0.2, with the increase in bending deformation, its bending load reaches the maximum value of 29.2 N at the bending deformation of 17.2 mm. For specimen R-0.4, the increase in the bending load is slower than the former, but it reaches the maximum bending load at a smaller bending deformation, reaching the maximum value of 25.1 N in a bending deformation of 17.3 mm. After reaching the maximum bending load, under the same bending deformation, the bending load of the SSMC with a unit cell aspect ratio of 0.4 becomes smaller than that of the aspect ratio 0.2. This indicates that the SSMC with the unit cell aspect ratio of 0.4 exhibits better bending deformation ability.

From the force–displacement curves of the SSMC with diamond kirigami structures of different aspect ratios shown in Figure 2b, it can be seen that there is a significant difference in the variation of bending load with bending deformation. For specimen D-0.2, the variation of bending load with bending deformation is relatively large, and the bending load reaches the maximum value of 42 N when the bending deformation is 18.1 mm. Subsequently, the bending deformation continues to increase while the bending load gradually decreases. The variation of bending load endured by specimen D-0.4 with bending deformation is relatively small, and the value of bending load reaches the maximum value of 34 N when the bending deformation is 20.2 mm. Subsequently, the bending load begins to decrease with the increase in bending deformation, and the bending load of specimens D-0.2 and D-0.4 decreases at a similar rate. Therefore, similar to the SSMC with the rectangular kirigami structure, the bending load capacity gradually decreases with the increase in the width-to-height ratio of the diamond cell, which will be reflected in the reduction in the maximum bending load. Specimen D-0.4 exhibits the highest bending deformation ability, which can produce more displacement under smaller bending loads, and its maximum bending load corresponds to larger bending deformation compared to specimen D-0.2.

From the force–displacement curves of the SSMC with elliptical kirigami structures of different aspect ratios shown in Figure 2b, the analysis shows that the maximum bending load of specimen E-0.2 reaches a maximum value of 33.3 N when the bending deformation is 19.8 mm. Subsequently, the bending load gradually decreases as the bending deformation increases. For specimen E-0.4, during the initial loading stage, the change rate of bending load with bending deformation is higher than that of specimen E-0.2. However, as the bending deformation increases, the change rate of the bending load decreases gradually and reaches the maximum bending load value of 27.6 N when the bending deformation is 20.3 mm. The maximum bending load is lower than that of specimen E-0.2, and then the bending load decreases faster. Therefore, compared with the SSMC of elliptical kirigami structures with smaller aspect ratios, the bending load capacity of larger aspect ratio decreases, but the bending deformation capacity increases. As a result, the superhydrophobic shape memory composite based on the diamond structure has the strongest bending load capacity, while the rectangular and elliptical structures have similar bending load capacity. In addition, further analysis based on the force–displacement curve showed that when the deformation is large, a larger aspect ratio can cause the component to undergo greater bending deformation under smaller bending loads, and a smaller aspect ratio can make the component have better bending load capacity. It can be concluded that a rectangular structure with an aspect ratio of 0.4 can produce greater displacement under smaller forces and show better bending deformation ability compared with other structures.

### 3.3. Surface Morphology Observation

**Figure 3:**
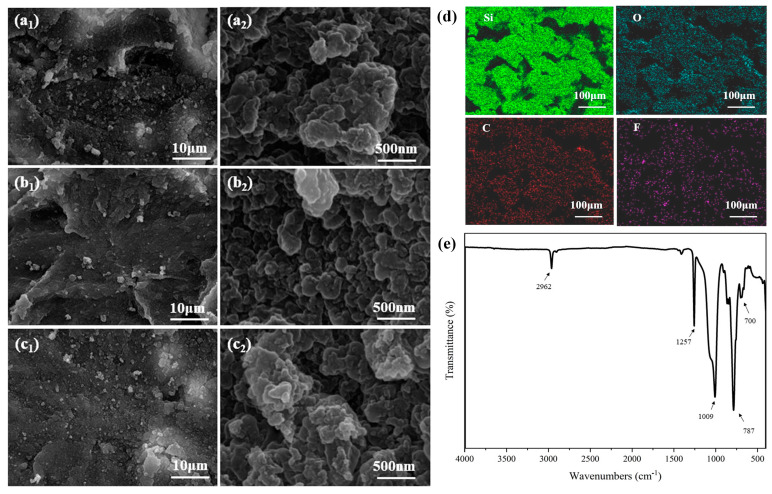
SEM images of superhydrophobic shape memory composites on (**a1**,**a2**) plane, (**b1**,**b2**) curve R = 6 mm, and (**c1**,**c2**) curve R = 8 mm, respectively. (**d**,**e**) are the EDS elemental map and FTIR spectrum of plane superhydrophobic shape memory composites, respectively.

The morphology of SSMC in plane and curve mode was observed using a scanning electron microscope. As shown in Figure 3a, it can be observed that the morphology of SSMC with different curvature radii is basically the same. When the magnification is increased to 5000 times, many papillae structures are found on the superhydrophobic functional surface. From the observation at higher magnification, it can be seen that a large number of nanoscale protrusions are distributed over multiple papillae structures, which significantly improves the air storage capacity and is one of the basic features to achieve superhydrophobicity. Therefore, the analysis showed that the change in the radius of curvature does not have a significant effect on the morphology of SSMC, which provides one of the prerequisites for the preparation of stable and excellent superhydrophobic functional surfaces. In addition, surface roughness plays a significant role in surface wettability. Three-dimensional optical microscopy was conducted on the SSMC plane, and the surface roughness was determined. As shown in Figure S2, it can be seen that there are traces of laser micromachining treatment on the SSMC, resulting in a rough surface. The average roughness (Ra) is 12.63 μm, and the root mean square roughness (Rq) is 18.91 μm. This further indicates that the SSMC has a rough surface, which is a prerequisite for achieving superhydrophobic performance.

Chemical composition is another prerequisite for superhydrophobic surfaces. From the EDS spectra, it can be seen that the elements of Si, O, C, and F were detected on the as-prepared superhydrophobic SMPC surface. In addition, FTIR was used to analyze the functional groups on the surface of SSMC in the scanning range of 400–4000 cm^−1^. From the FTIR spectrum shown in Figure 3e, the absorption peaks at 2962 cm^−1^, 1257 cm^−1^, and 700 cm^−1^ are due to the stretching vibration, symmetric bending vibration, and in-plane bending vibration of Si-CH_3_ on the silicone rubber [28,29]. The peak at 700 cm^−1^ is caused by the C-F bond, indicating the successful fluorination reaction of multi-walled carbon nanotubes. The absorption peak at 1009 cm^−1^ is due to the stretching vibration of the Si-O-Si bond formed by the PFOTS reaction [30,31]. Therefore, it can be confirmed that CNTs have been successfully modified through PFOTS and mixed with silicone rubber to present superhydrophobic properties. The water contact angle is an important parameter to describe the static wetting performance of the SSMC, and the rolling angle is a parameter to characterize the water adhesion of SSMC. As shown in Figure S3a, the average water contact angle on the superhydrophobic surface is 156.9 ± 4.4°. Also, the rolling angle on the SSMC surface is 3 ± 0.5°. Therefore, the SSMC surface provided good superhydrophobic and low-adhesion performance.

### 3.4. Investigation of Droplet Impacting Performance

**Figure 4:**
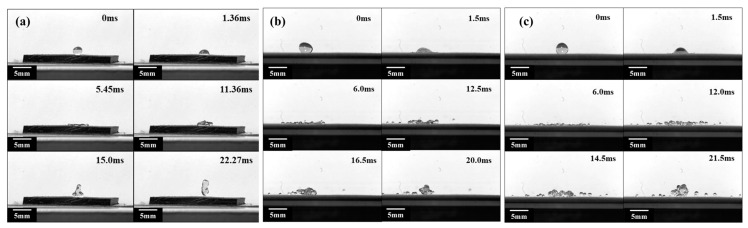
Droplets impacting on the plane superhydrophobic shape memory composites at the height of (**a**) 50 mm, (**b**) 100 mm, and (**c**) 150 mm.

To describe the droplet impact phenomenon, this study also includes the analysis of the evolution of the droplet impact state. As shown in Figure 4a, the droplet was released at a height of 5 cm (with impact velocity of 0.99 m/s). When a droplet contacts a superhydrophobic surface, it is pressurized and generates capillary waves at 1.36 ms, and the capillary waves propagate from the contact point toward the top of the droplet. At the same time, under the stretching effect of the inertial force, the droplet undergoes a spreading process in all directions. As the droplet continues to spread, due to the lateral stretching effect, the upper part of the droplet collapses and creates a pit, and immediately the droplet reaches its maximum spreading diameter. Due to the squeezing effect of the central depression around the droplet, the droplet converges from all sides to the middle, where it collides and generates a jet at 11.36 ms. The liquid droplet rebounds upwards from the impact center at 15 ms, and its shape quickly retracts from a pie-like shape at maximum spreading to a cylindrical shape at 19.5 ms. Subsequently, the droplets rebound and separate from the superhydrophobic horizontal plane at 22.27 ms. Throughout the entire process, the droplets are divided into three evolution stages: the spreading stage, retraction stage, and rebound stage, and the droplets completely bounce off the surface, indicating that the superhydrophobic horizontal plane has anti-wetting performance. By changing the height of the droplets, the droplets hit the superhydrophobic surface with different velocities, and the state of the droplets changed drastically. Figure 4b,c show the impact phenomenon of liquid droplets that fell from heights of 100 mm and 150 mm (with an impact velocity of 1.40 m/s and 1.72 m/s, respectively) on superhydrophobic surfaces. It is found that the droplet evolution process is similar in the two cases. After the droplet contacts the superhydrophobic surface, the droplet rapidly spreads around under the influence of inertial force. Due to the strong effect of the inertial force on the edge of the droplet, the surface tension of the droplet is not enough to bind the microdroplets at the edge. This leads to multiple fractures between the microdroplets around and the main part of the droplet, resulting in many “finger-like” droplets at 6 ms in Figure 4b,c. During the retraction process, due to the effect of surface tension, the main droplets quickly retract, and more microdroplets at the edges are pulled apart to form secondary droplets The main part of the liquid droplet experiences a rebound phenomenon.

**Figure 5:**
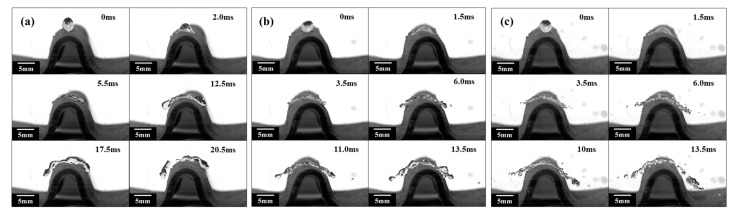
Droplet impacts on curved superhydrophobic shape memory composites (R = 6 mm) at the height of (**a**) 50 mm, (**b**) 100 mm, and (**c**) 150 mm.

The droplet impacting test was also conducted on the SSMC surface with a radius of 6 mm, as shown in Figure 5. When a droplet falls from a height of 50 mm and impacts a superhydrophobic surface with a radius of 6 mm with an initial velocity of 0.99 m/s, the droplet spreads rapidly and creates capillary waves at the bottom of the droplet then propagates from the bottom of the droplet to the top of the droplet, as shown in Figure 5a. Under the stretching effect of inertial force along the curved surface, the droplets continued to spread, forming a liquid film in the middle and a “liquid ring” at the outermost edge. The “liquid ring” continuously gathered to form a larger “liquid ring” volume at the outermost side, reaching the maximum spreading state. Afterwards, the droplets retracted and eventually experienced a cake-like bounce, bouncing off the superhydrophobic surface at 17.5 ms. After bouncing off the superhydrophobic surface, the droplets showed a morphing shape in the middle, and the surface tension was not enough to bind the droplets. This led to a fracture at 20.5 ms, whereupon the two parts of the droplets continued to rebound. When a droplet was released from a height of 100 mm or 150 mm, the impact velocity on the 6 mm radius SSMC surface was 1.40 m/s and 1.72 m/s, respectively, as shown in Figure 5b,c. This shows that after the droplet came into contact with the superhydrophobic surface, the inertia force was much greater than the surface tension, which led to the rapid spreading process of the droplet, and no capillary waves were observed during this process at 1.5 ms. As the spreading process continued, many “finger-like” droplets were generated at the edge of the droplets and curled up. More “finger-like” droplets then broke off, forming many secondary small droplets at 6 ms. Consequently, the droplet splashed at 10 ms and gradually fragmented at 13.5 ms.

**Figure 6:**
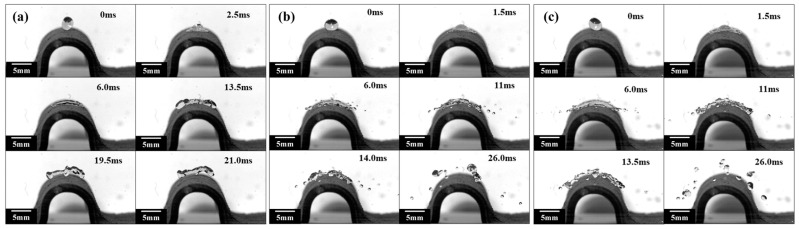
Droplet impacts on curved superhydrophobic shape memory composites (R = 8 mm) at the height of (**a**) 50 mm, (**b**) 100 mm, and (**c**) 150 mm.

As shown in Figure 6, the experimental results of liquid droplets falling from different heights on SSMC surfaces with an impact radius of 8 mm are presented. When a droplet is released from a height of 50 mm and collides with a superhydrophobic surface of 8 mm radius with an initial velocity of 0.99 m/s, the capillary waves are generated at the bottom of the droplet due to the squeezing effect of the superhydrophobic surface. Afterward, the capillary waves propagate from the bottom of the droplet to the top of the droplet at 2.5 ms. Under the stretching effect of the inertial force along the surface, the droplets continue to spread, forming a liquid film on the inner side of the droplets and a “liquid ring” at the surrounding edges at 6.0 ms. The internal liquid film continues to spread outward and gathers at the outer edge of the droplets, forming a larger and thicker “liquid ring” on the outer side of the droplets. After reaching the maximum spreading diameter, the liquid droplet undergoes a cake-like bounce and completely detaches from the superhydrophobic surface at 19.5 ms. After the droplet bounces off the superhydrophobic surface, it still keeps the cake shape and rebounds. Because the initial impact velocity of the droplet is low and the radius of the superhydrophobic surface is large, the droplet does not break at 21 ms.

When a droplet is released from a height of 100 mm or 150 mm, it rapidly spreads along the curved surface and generates “finger-like” droplets at the edge of the droplet under the influence of inertial force at 1.5 ms, as shown in Figure 6b,c. As the droplets continue to spread outward, the “finger-like” droplets detach from the main part of the droplets and splatter, forming a larger and thinner liquid film in the middle of the droplets at 6 ms. Due to insufficient surface tension to bind the liquid film, the liquid film breaks at many necking positions and bounces off the superhydrophobic surface at 11 ms. After bouncing off the superhydrophobic surface, the droplets continue to fracture and break and eventually undergo a rebound process in the form of many small droplets at 26 ms.

Based on the above analysis, it is evident that there are significant differences in the impact phenomenon when impacting on plane SSMC surfaces with or SSMC surfaces with different curvature radii. When the droplet impacts the superhydrophobic horizontal plane at a lower velocity, the droplet does not break and undergoes a complete process of impact, including spreading, retraction, and rebound. When the droplet collides with the curved superhydrophobic surface at a lower velocity, it undergoes droplet impact, spreading, retraction, and a cake-like rebound process. However, both the superhydrophobic horizontal plane and the superhydrophobic surface undergo droplet spreading, fragmentation, and splashing processes at higher impact velocities. The difference is that when a droplet collides with a superhydrophobic horizontal plane, the “finger-like” droplet is splashed at the edge of the droplet, causing the main central droplet to retract and rebound, bouncing off the superhydrophobic horizontal plane. For curved superhydrophobic surfaces, the droplets undergo a cake-like rebound and continue to undergo a crushing process, ultimately bouncing off the superhydrophobic surface in the form of many small droplets. Therefore, when impacting the surface at a higher velocity, the contact time of the droplet is shorter, reducing the impact of the droplet on the superhydrophobic surface.

**Figure 7:**
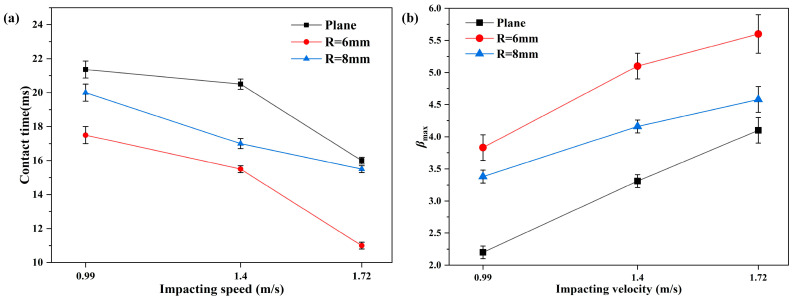
(**a**) The relationship between the droplet contact time and impact velocity; (**b**) the relationship between the maximum spreading coefficient of droplets and impact velocity.

Furthermore, it can be seen from Figure 7a that when a droplet collides at different velocities, the contact time of the droplet decreases with the increase in the impact velocity. At the same impact velocity, the contact time of the droplet decreases with the decrease in the radius of curvature. When the droplet collides with the superhydrophobic horizontal surface at 0.99 m/s, the contact time is 21.36 ms. But when the droplet collides with the superhydrophobic curved surface with a curvature radius of 6 mm at velocity of 1.72 m/s, the contact time is reduced to 11 ms (i.e., a reduction of about 48.5%). Therefore, according to the analysis, increasing the curvature of the superhydrophobic surface or increasing the impact velocity of the droplet reduces the contact time between the droplet and the superhydrophobic surface and thus reduces the impact of the droplet on the superhydrophobic surface.

Moreover, the maximum spreading coefficient (*β_max_*) represents the ratio of the spreading diameter *d_max_* at the maximum spreading diameter to the initial diameter *d*_0_, which is:

(3) $$\beta_{\max}=\frac{d_{\max}}{d_{0}}$$

As shown in Figure 7b, it can be concluded that the *β_max_* increases with the increase in impact velocity. When a droplet impacts on the SSMC surface with different curvatures at the same velocity, the *β_max_* increases continuously with the increase in curvature. Moreover, it can be seen that when the droplet collides on the plane SSMC surface at a velocity of 0.99 m/s, the *β_max_* is the smallest, at about 2.2. But, impacting on the SSMC surface with a curvature radius of 6mm at an impacting velocity of 1.72 m/s, the *β_max_* is the largest, at about 5.6. In summary, the increase in *β_max_* should be attributed to the increase in inertial force from different release heights, and the decrease in curvature radius leads to a decrease in the contact area between the droplet and the SSMC surface.

**Figure 8:**
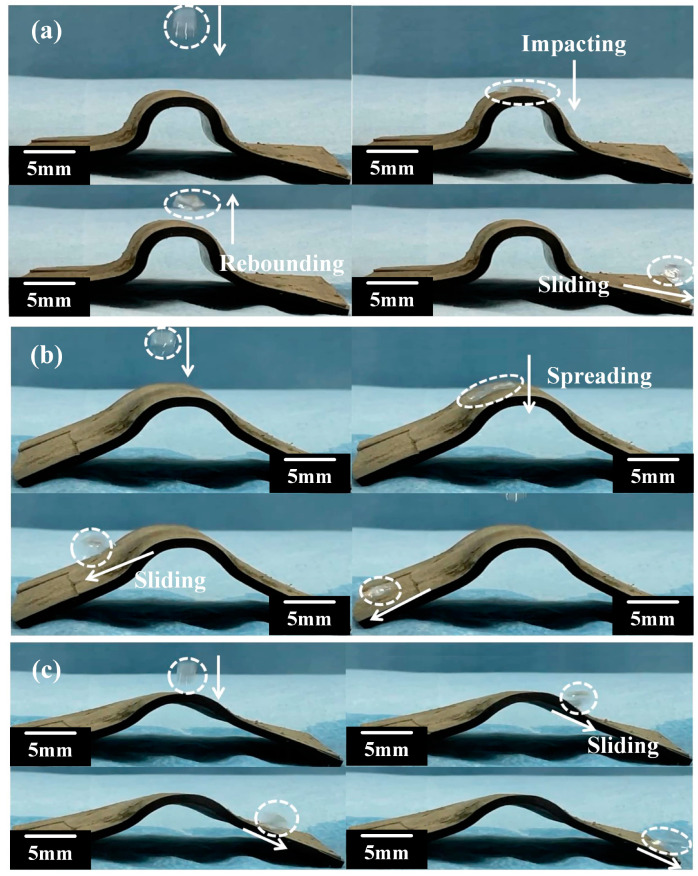
Uniform wetting performance of superhydrophobic shape memory composites at the times of (**a**) 18 s, (**b**) 72 s, and (**c**) 80 s.

To further investigate the wetting performance during the shape recovery process of SSMC, the sample was placed on a heating table of 180 °C, and a needle tube was used to generate droplets to impact the SSMC surface. As shown in Figure 8a–c, the droplets were released at 18 s, 72 s, and 80 s, respectively. It can be seen that during the shape memory recovery process, the droplets collide with the superhydrophobic surface and completely rebound, without producing secondary droplets or adhering to the superhydrophobic surface. Finally, the droplets completely bounce off the superhydrophobic functional surface. This indicates that the SSMC surface maintained anti-wetting properties during the shape memory recovery process.

## 4. Conclusions

In this study, we prepared shape memory epoxy resin and its composite materials, with glass transition temperatures of 88 °C and 97 °C, respectively. The corresponding shape recovery rates were 92.3% and 98.1%. Based on the kirigami design, superhydrophobic shape memory composite (SSMC) was prepared by compounding composite materials with superhydrophobic CNT@PDMS, followed by laser ablation. The water contact angle of SSMC is 156.9 ± 4.4°, while the rolling angle is 3 ± 0.5°. Also, its shape recovery rate is about 85.11%. Among the various kirigami structures considered herein, the diamond structure has the strongest bending load capacity, and the larger aspect ratio can give the structure better bending deformation capacity. Also, the rectangular structure with an aspect ratio of 0.4 has the best bending deformation capacity. The anti-wetting performance of the material was studied through droplet impact tests on superhydrophobic surfaces. It can be concluded that the droplets completely rebound on the SSMC surface at low impact velocity, but the droplets break and rebound at high impact velocity. In addition, the contact time decreases with the increase in impact velocity, and the maximum spreading coefficient increases with the increase in impact velocity. For the SSMC surface with different radius of curvature, the contact time of droplets is greatly shortened. Moreover, the contact time decreases with the decrease in the radius of curvature, and the maximum spreading coefficient increases with the decrease in the radius of curvature. The experimental results indicate that the surface of the prepared SSMC provides good anti-wetting performance. Consequently, the SSMC is expected to be applied to deformable wing skin structures to solve problems such as droplet impact and adhesion on the skin surface.

## Data Availability

Most of the datasets supporting the conclusions of this article are included within this article. The datasets used or analyzed during the current study can be shared with permission from the corresponding author upon reasonable request.

## Supplementary Materials

The following supporting information can be downloaded at: https://www.mdpi.com/article/10.3390/polym15183738/s1, Figure S1: Schematic diagram of the impact test device; Figure S2: 3D optical microscope images of superhydrophobic shape memory composite materials; Figure S3: Wettability on superhydrophobic shape memory composite: (a) water contact angle, (b) water sliding angle; Table S1: Parameters of each kirigami cell; Table S2: Parameters of droplets impacting test.
