# Supplementary material for: Design of Superhydrophobic Shape Memory Composites with Kirigami Structures and Uniform Wetting Property
Source: Polymers (Basel). 2023 Sep 12;15(18):3738. doi: 10.3390/polym15183738 (PMC10536611; doi:10.3390/polym15183738)
Supplement: Supplementary file 1 [file polymers-15-03738-s001.zip › polymers-2588017-supplementary.pdf]

Design of superhydrophobic shape memory composites with kirigami structures and uniform wetting property

Zhe Zhao[a], Xinlin Li[a*], Dongsong Wei[b], Jian Sun[a*], Jinsong Leng[a]

a. Centre for Composite Materials and Structures, Harbin Institute of Technology (HIT), Harbin, 150080, PR China

b. Key Laboratory of Bionic Engineering (Ministry of Education), Jilin University, Changchun, 130022, PR China

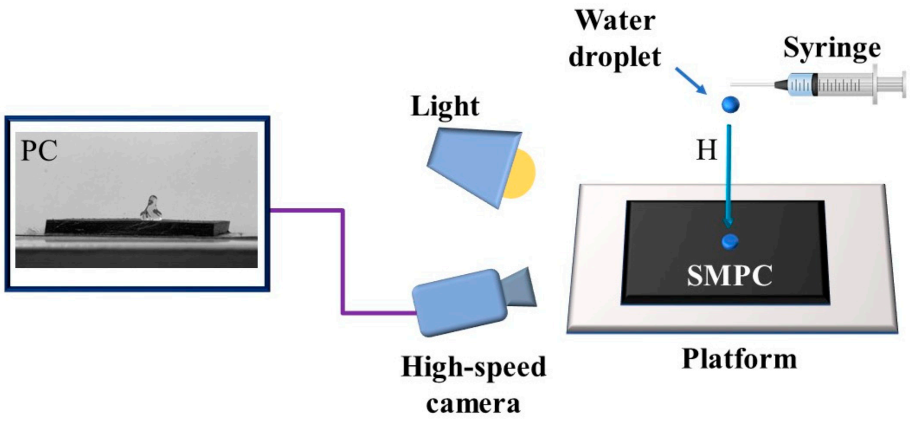

Figure S1 Schematic diagram of the impact test device

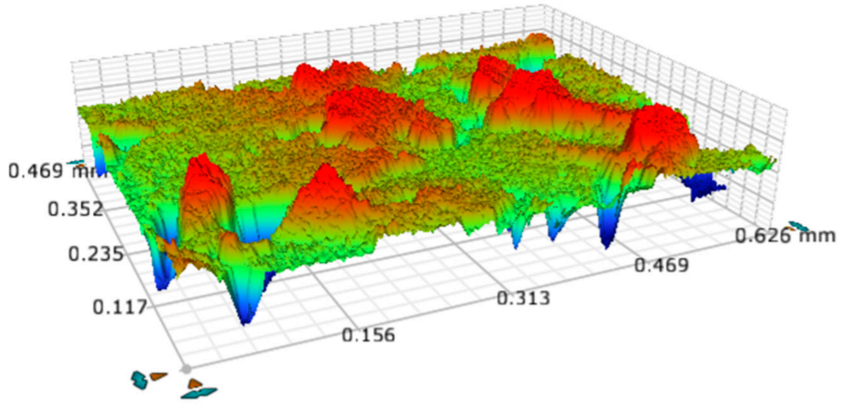

Figure S2 3D optical microscope images of superhydrophobic shape memory composite materials

* Corresponding authors: Sunjian@hit.edu.cn, lixinlin@hit.edu.cn

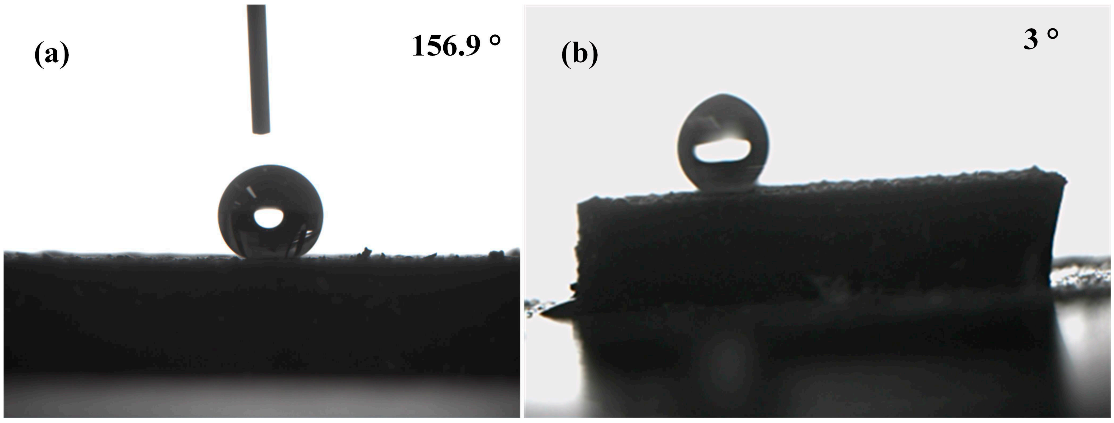

Figure S3 Wettability on superhydrophobic shape memory composite: (a) water contact angle, (b) water sliding angle.

Table S1 Parameters of each kirigami cell

Cell	h(mm)	b(mm)	Aspect ratio	Samples
Rectangular	10	2	0.20	R-0.2
	10	4	0.40	R-0.4
Diamond	10	2	0.20	D-0.2
	10	4	0.40	D-0.4
Elliptical	10	2	0.20	E-0.2
	10	4	0.40	E-0.4

Table S2 Parameters of droplets impacting test

Samples	Height(mm)	Impacting speed(m/s)
Plane-SSMC	50	0.99
	100	1.40
	150	1.72
Curve（R=6mm）	50	0.99
	100	1.40
	150	1.72
Curve（R=8mm）	50	0.99
	100	1.40
	150mm	1.72m/s
